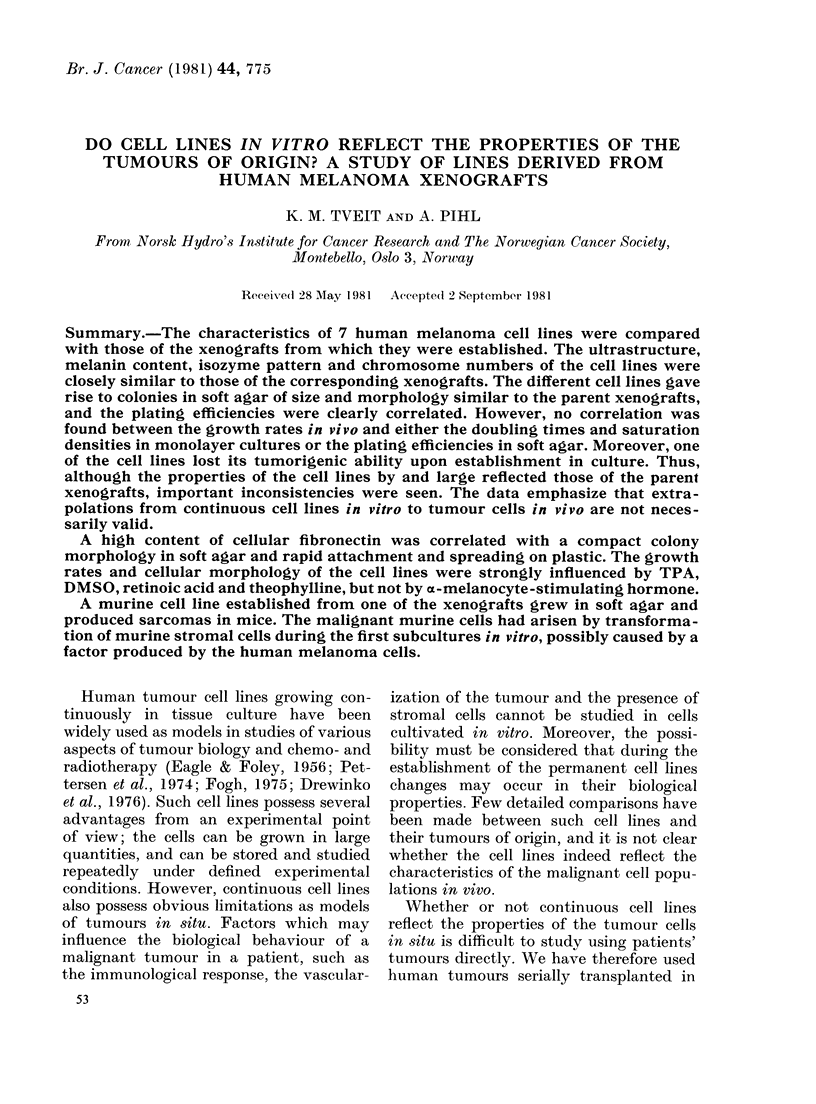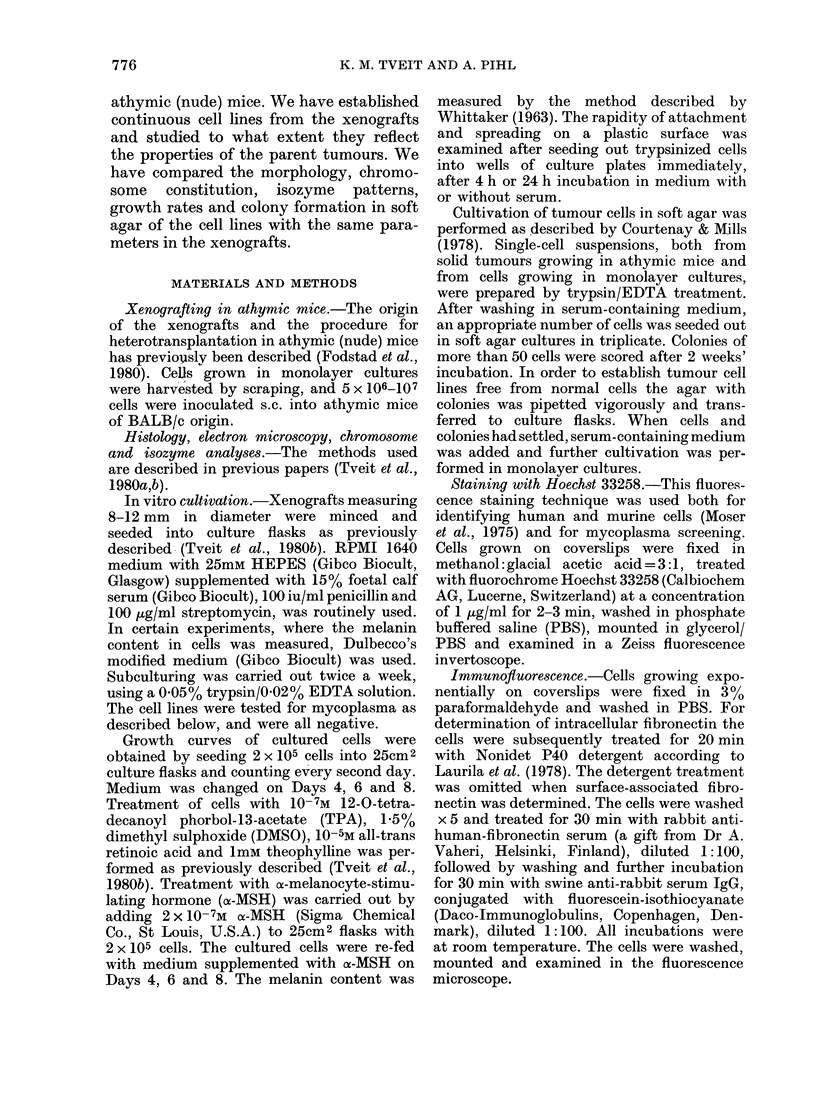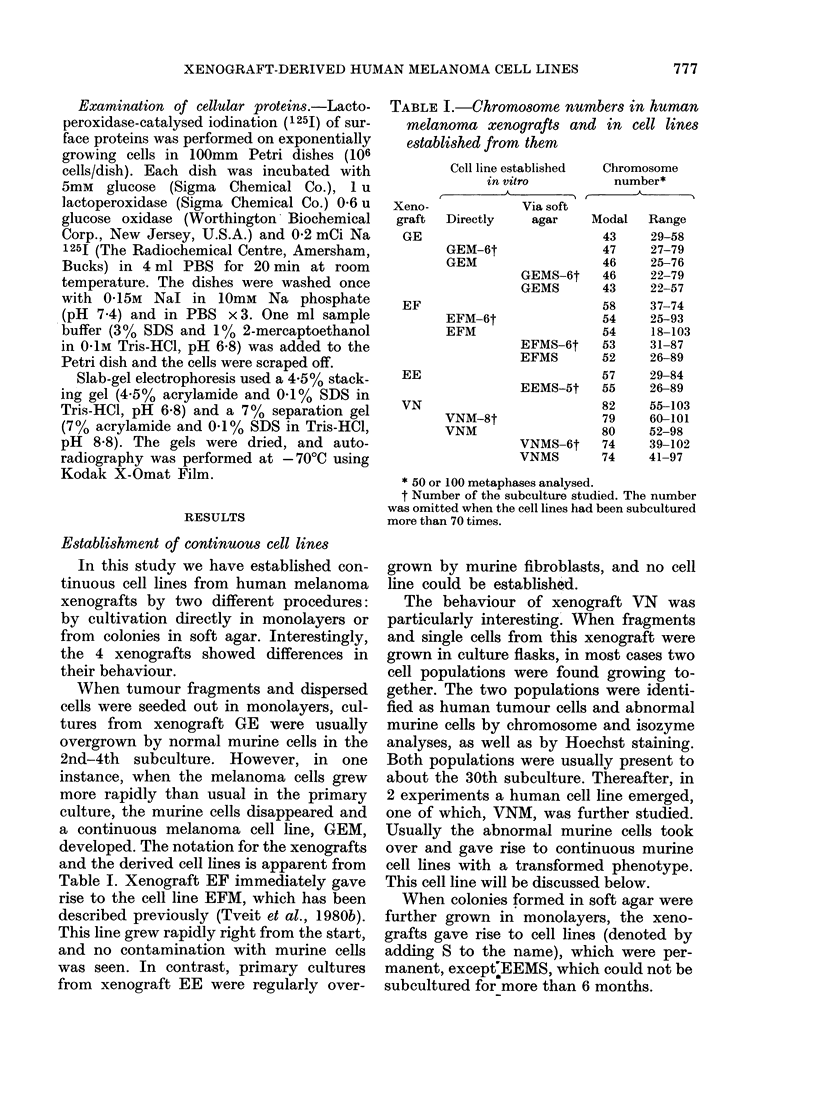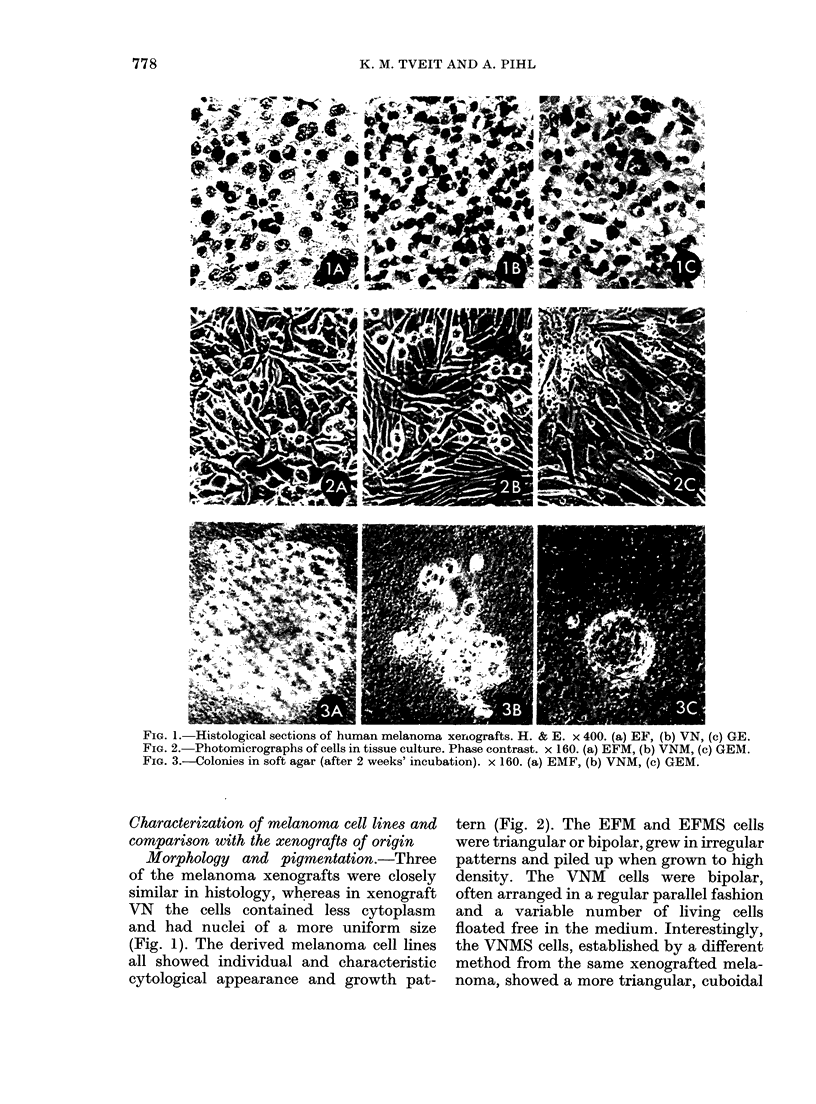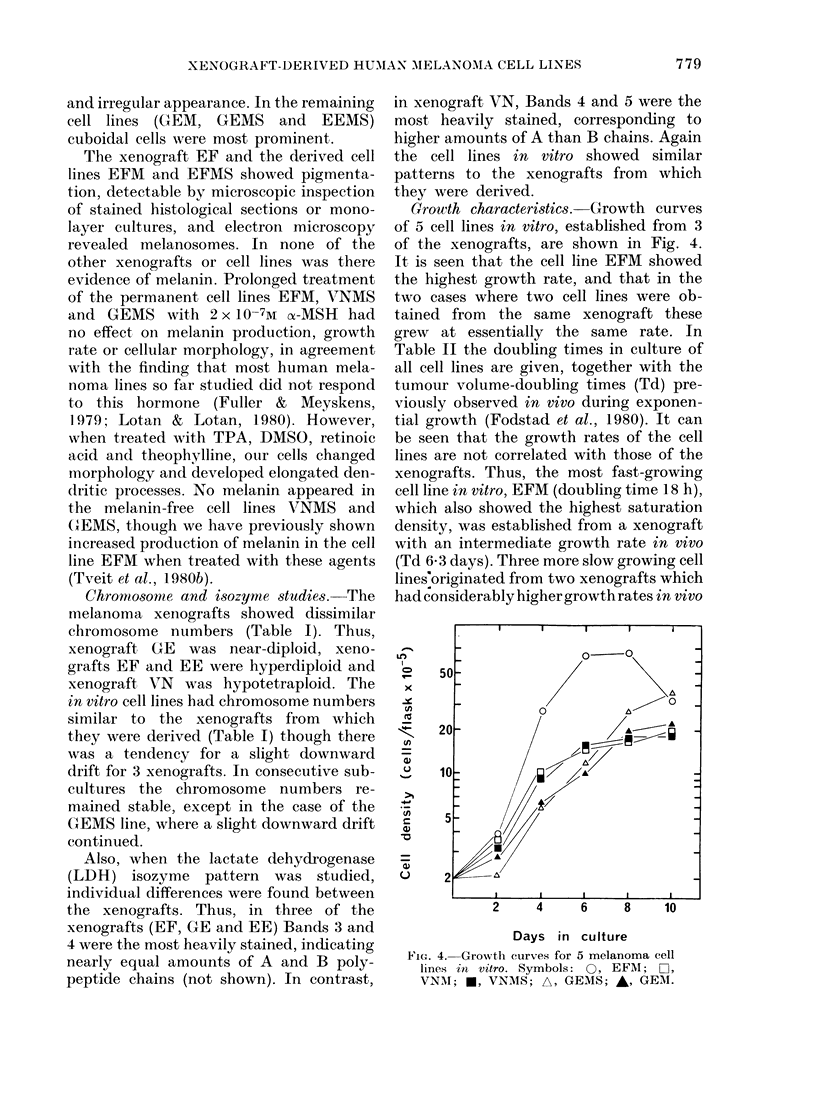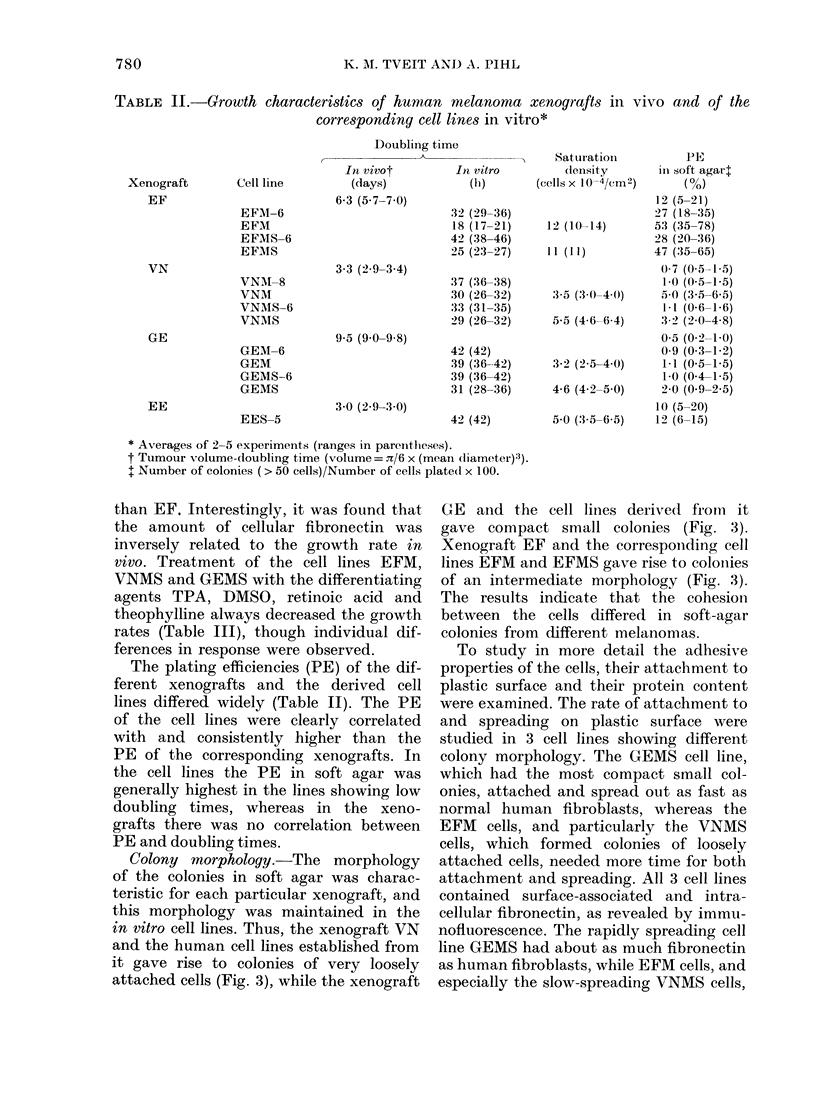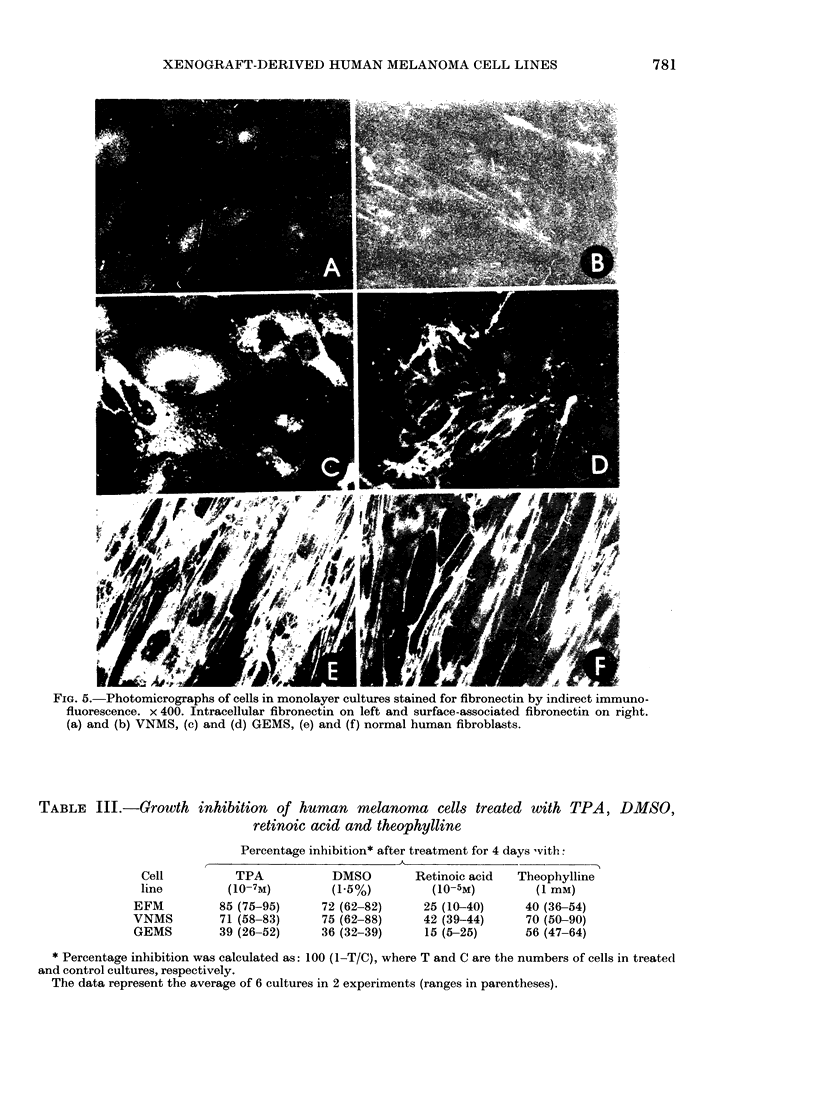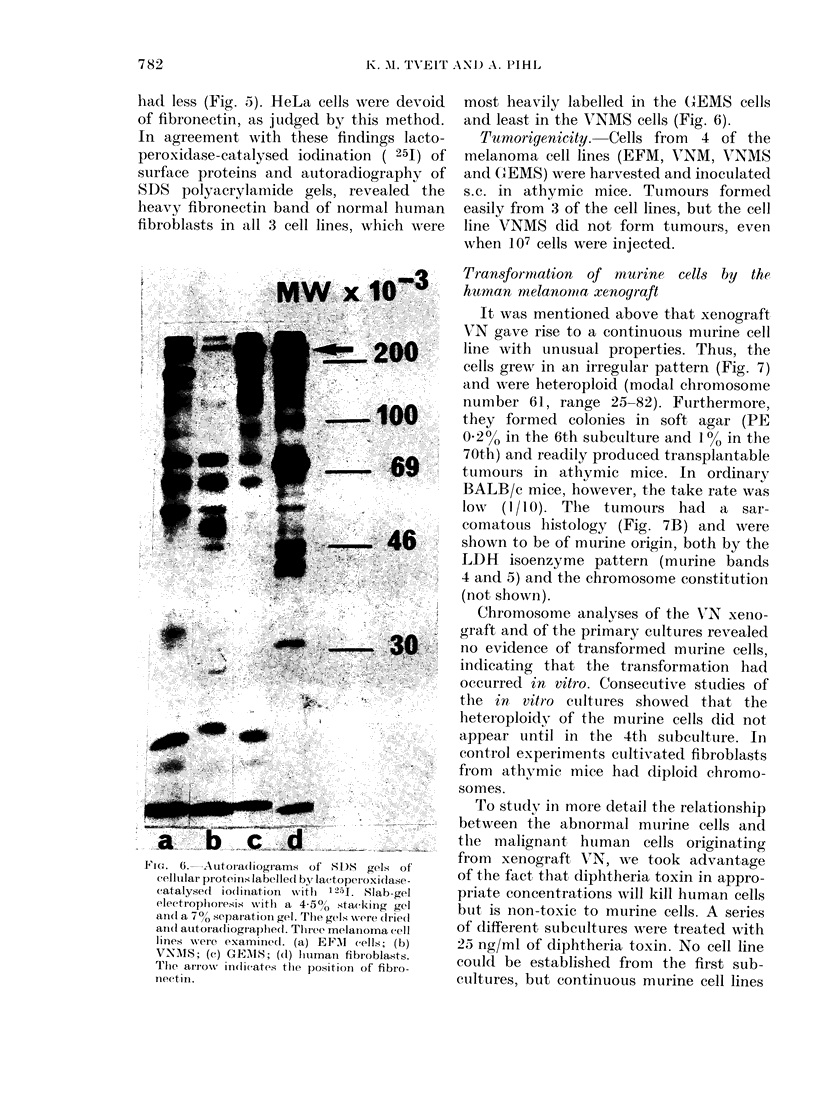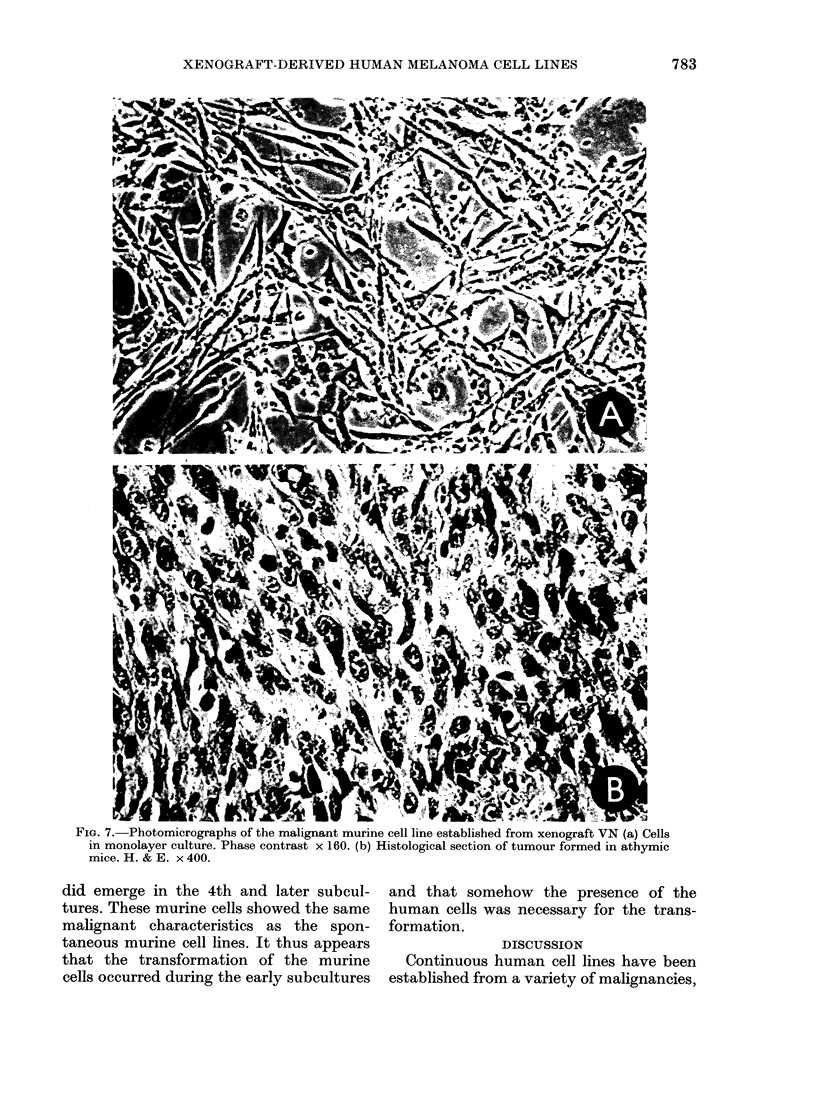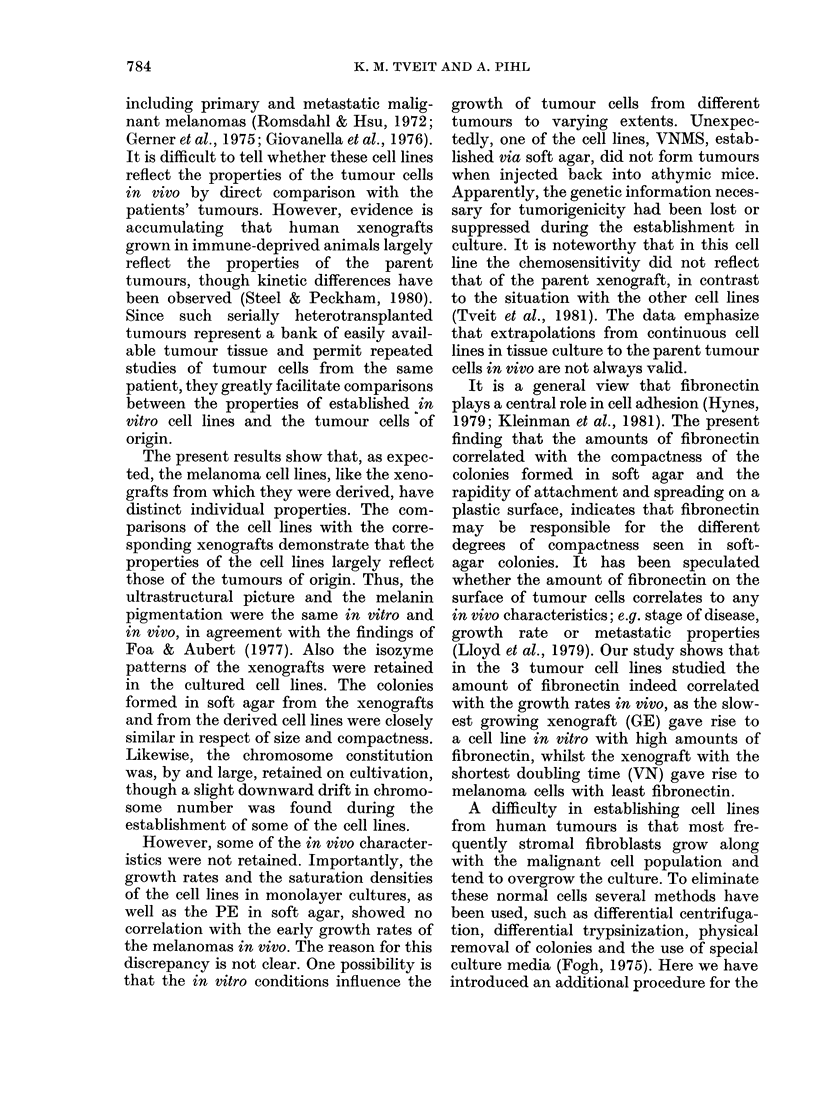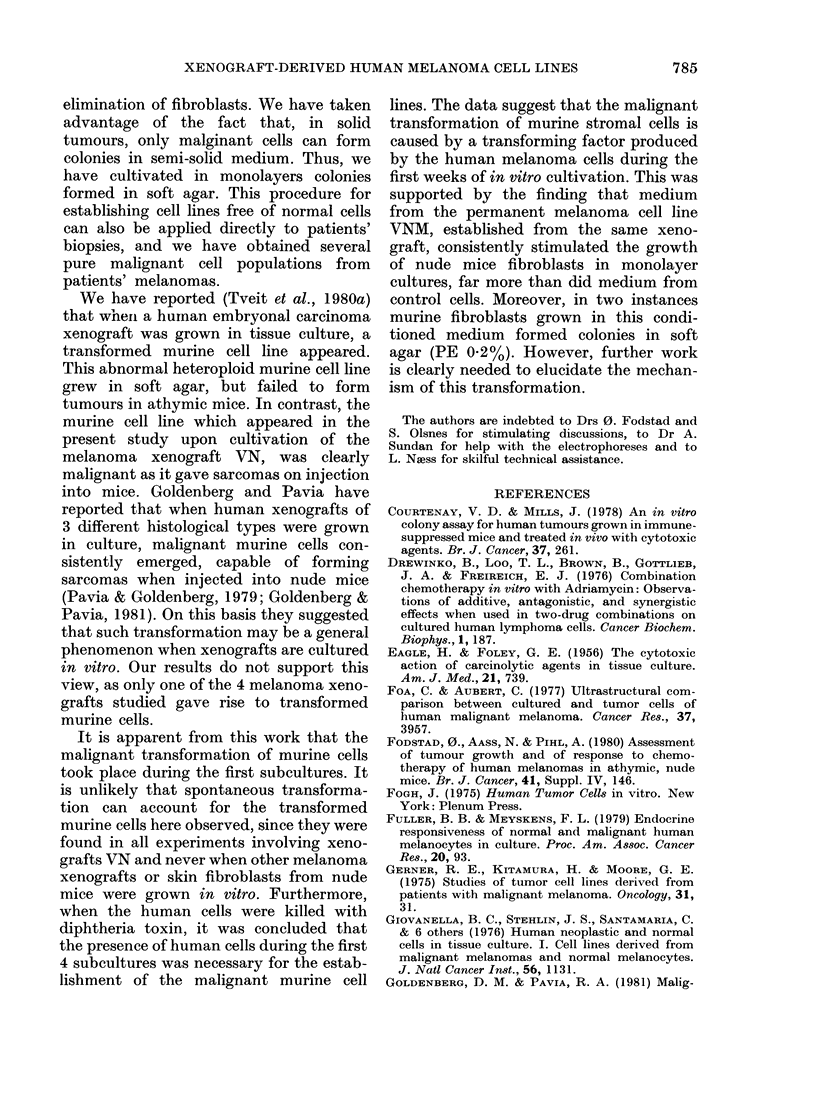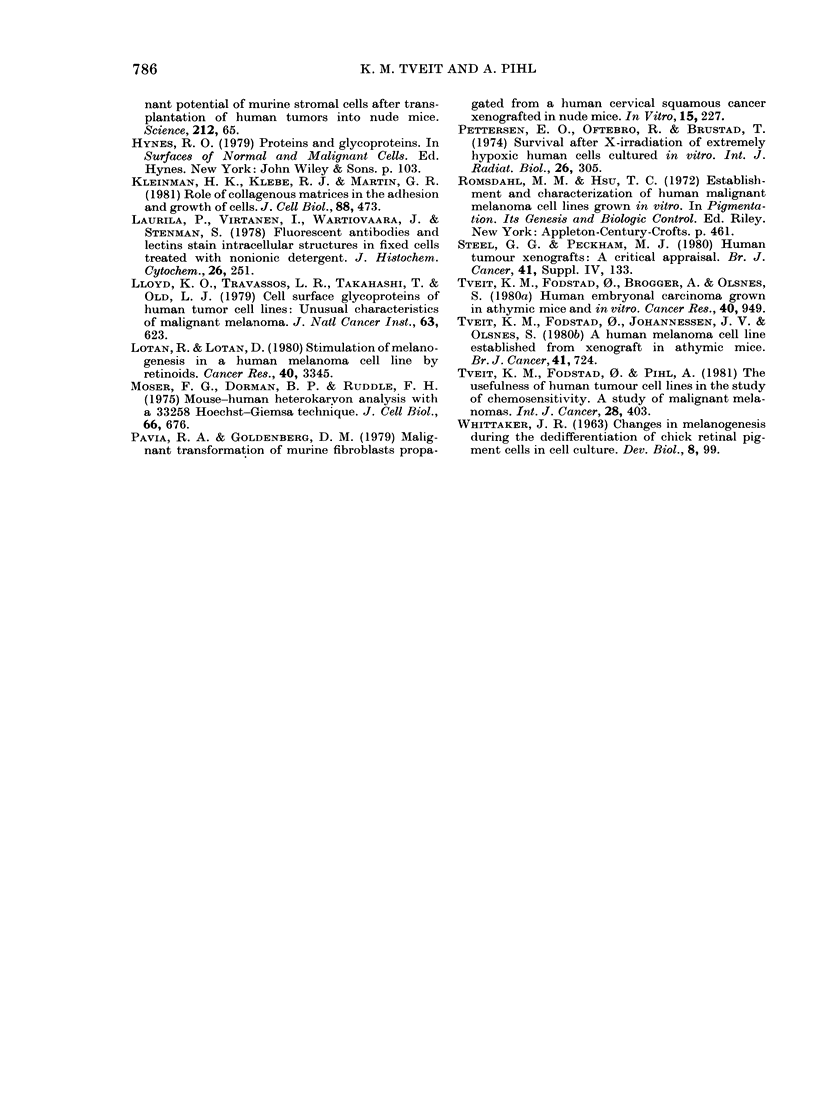# Do cell lines in vitro reflect the properties of the tumours of origin? A study of lines derived from human melanoma xenografts.

**DOI:** 10.1038/bjc.1981.276

**Published:** 1981-12

**Authors:** K. M. Tveit, A. Pihl

## Abstract

**Images:**


*Br. J. Cancer* (1981) **44**, 775

### DO CELL LINES *IN VITRO* REFLECT THE PROPERTIES OF THE TUMOURS OF ORIGIN? A STUDY OF LINES DERIVED FROM HUMAN MELANOMA XENOGRAFTS

K. M. TVEIT AND A. PIHL

*From Norsk Hydro's Institute for Cancer Research and The Norwegian Cancer Society, Montebello, Oslo 3, Norway*

Received 28 May 1981 Accepted 2 September 1981

**Summary.—The characteristics of 7 human melanoma cell lines were compared with those of the xenografts from which they were established. The ultrastructure, melanin content, isozyme pattern and chromosome numbers of the cell lines were closely similar to those of the corresponding xenografts. The different cell lines gave rise to colonies in soft agar of size and morphology similar to the parent xenografts, and the plating efficiencies were clearly correlated. However, no correlation was found between the growth rates *in vivo* and either the doubling times and saturation densities in monolayer cultures or the plating efficiencies in soft agar. Moreover, one of the cell lines lost its tumorigenic ability upon establishment in culture. Thus, although the properties of the cell lines by and large reflected those of the parent xenografts, important inconsistencies were seen. The data emphasize that extrapolations from continuous cell lines *in vitro* to tumour cells *in vivo* are not necessarily valid.**

**A high content of cellular fibronectin was correlated with a compact colony morphology in soft agar and rapid attachment and spreading on plastic. The growth rates and cellular morphology of the cell lines were strongly influenced by TPA, DMSO, retinoic acid and theophylline, but not by α-melanocyte-stimulating hormone.**

**A murine cell line established from one of the xenografts grew in soft agar and produced sarcomas in mice. The malignant murine cells had arisen by transformation of murine stromal cells during the first subcultures *in vitro*, possibly caused by a factor produced by the human melanoma cells.**

Human tumour cell lines growing continuously in tissue culture have been widely used as models in studies of various aspects of tumour biology and chemo- and radiotherapy (Eagle & Foley, 1956; Pettersen *et al.*, 1974; Fogh, 1975; Drewinko *et al.*, 1976). Such cell lines possess several advantages from an experimental point of view; the cells can be grown in large quantities, and can be stored and studied repeatedly under defined experimental conditions. However, continuous cell lines also possess obvious limitations as models of tumours *in situ*. Factors which may influence the biological behaviour of a malignant tumour in a patient, such as the immunological response, the vascularization of the tumour and the presence of stromal cells cannot be studied in cells cultivated *in vitro*. Moreover, the possibility must be considered that during the establishment of the permanent cell lines changes may occur in their biological properties. Few detailed comparisons have been made between such cell lines and their tumours of origin, and it is not clear whether the cell lines indeed reflect the characteristics of the malignant cell populations *in vivo*.

Whether or not continuous cell lines reflect the properties of the tumour cells *in situ* is difficult to study using patients' tumours directly. We have therefore used human tumours serially transplanted in athymic (nude) mice. We have established continuous cell lines from the xenografts and studied to what extent they reflect the properties of the parent tumours. We have compared the morphology, chromosome constitution, isozyme patterns, growth rates and colony formation in soft agar of the cell lines with the same parameters in the xenografts.

### MATERIALS AND METHODS

*Xenografting in athymic mice.*—The origin of the xenografts and the procedure for heterotransplantation in athymic (nude) mice has previously been described (Fodstad *et al.*, 1980). Cells grown in monolayer cultures were harvested by scraping, and $5 \times 10^6$–$10^7$ cells were inoculated s.c. into athymic mice of BALB/c origin.

*Histology, electron microscopy, chromosome and isozyme analyses.*—The methods used are described in previous papers (Tveit *et al.*, 1980*a,b*).

In vitro *cultivation.*—Xenografts measuring 8–12 mm in diameter were minced and seeded into culture flasks as previously described (Tveit *et al.*, 1980*b*). RPMI 1640 medium with 25mM HEPES (Gibco Biocult, Glasgow) supplemented with 15% foetal calf serum (Gibco Biocult), 100 iu/ml penicillin and 100 $\mu$g/ml streptomycin, was routinely used. In certain experiments, where the melanin content in cells was measured, Dulbecco's modified medium (Gibco Biocult) was used. Subculturing was carried out twice a week, using a 0·05% trypsin/0·02% EDTA solution. The cell lines were tested for mycoplasma as described below, and were all negative.

Growth curves of cultured cells were obtained by seeding $2 \times 10^5$ cells into 25cm$^2$ culture flasks and counting every second day. Medium was changed on Days 4, 6 and 8. Treatment of cells with $10^{-7}$M 12-O-tetradecanoyl phorbol-13-acetate (TPA), 1·5% dimethyl sulphoxide (DMSO), $10^{-5}$M all-trans retinoic acid and 1mM theophylline was performed as previously described (Tveit *et al.*, 1980*b*). Treatment with $\alpha$-melanocyte-stimulating hormone ($\alpha$-MSH) was carried out by adding $2 \times 10^{-7}$M $\alpha$-MSH (Sigma Chemical Co., St Louis, U.S.A.) to 25cm$^2$ flasks with $2 \times 10^5$ cells. The cultured cells were re-fed with medium supplemented with $\alpha$-MSH on Days 4, 6 and 8. The melanin content was measured by the method described by Whittaker (1963). The rapidity of attachment and spreading on a plastic surface was examined after seeding out trypsinized cells into wells of culture plates immediately, after 4 h or 24 h incubation in medium with or without serum.

Cultivation of tumour cells in soft agar was performed as described by Courtenay & Mills (1978). Single-cell suspensions, both from solid tumours growing in athymic mice and from cells growing in monolayer cultures, were prepared by trypsin/EDTA treatment. After washing in serum-containing medium, an appropriate number of cells was seeded out in soft agar cultures in triplicate. Colonies of more than 50 cells were scored after 2 weeks' incubation. In order to establish tumour cell lines free from normal cells the agar with colonies was pipetted vigorously and transferred to culture flasks. When cells and colonies had settled, serum-containing medium was added and further cultivation was performed in monolayer cultures.

*Staining with Hoechst* 33258.—This fluorescence staining technique was used both for identifying human and murine cells (Moser *et al.*, 1975) and for mycoplasma screening. Cells grown on coverslips were fixed in methanol:glacial acetic acid = 3:1, treated with fluorochrome Hoechst 33258 (Calbiochem AG, Lucerne, Switzerland) at a concentration of 1 $\mu$g/ml for 2–3 min, washed in phosphate buffered saline (PBS), mounted in glycerol/PBS and examined in a Zeiss fluorescence invertoscope.

*Immunofluorescence.*—Cells growing exponentially on coverslips were fixed in 3% paraformaldehyde and washed in PBS. For determination of intracellular fibronectin the cells were subsequently treated for 20 min with Nonidet P40 detergent according to Laurila *et al.* (1978). The detergent treatment was omitted when surface-associated fibronectin was determined. The cells were washed ×5 and treated for 30 min with rabbit anti-human-fibronectin serum (a gift from Dr A. Vaheri, Helsinki, Finland), diluted 1:100, followed by washing and further incubation for 30 min with swine anti-rabbit serum IgG, conjugated with fluorescein-isothiocyanate (Daco-Immunoglobulins, Copenhagen, Denmark), diluted 1:100. All incubations were at room temperature. The cells were washed, mounted and examined in the fluorescence microscope.

*Examination of cellular proteins.*—Lactoperoxidase-catalysed iodination ($^{125}I$) of surface proteins was performed on exponentially growing cells in 100mm Petri dishes ($10^6$ cells/dish). Each dish was incubated with 5mM glucose (Sigma Chemical Co.), 1 u lactoperoxidase (Sigma Chemical Co.) 0·6 u glucose oxidase (Worthington Biochemical Corp., New Jersey, U.S.A.) and 0·2 mCi Na $^{125}I$ (The Radiochemical Centre, Amersham, Bucks) in 4 ml PBS for 20 min at room temperature. The dishes were washed once with 0·15M NaI in 10mM Na phosphate (pH 7·4) and in PBS ×3. One ml sample buffer (3% SDS and 1% 2-mercaptoethanol in 0·1M Tris-HCl, pH 6·8) was added to the Petri dish and the cells were scraped off.

Slab-gel electrophoresis used a 4·5% stacking gel (4·5% acrylamide and 0·1% SDS in Tris-HCl, pH 6·8) and a 7% separation gel (7% acrylamide and 0·1% SDS in Tris-HCl, pH 8·8). The gels were dried, and autoradiography was performed at −70°C using Kodak X-Omat Film.

TABLE I.—*Chromosome numbers in human melanoma xenografts and in cell lines established from them*

| Xenograft | Cell line established *in vitro*: Directly | Via soft agar | Chromosome number*: Modal | Range |
|---|---|---|---|---|
| GE | | | 43 | 29–58 |
| | GEM-6† | | 47 | 27–79 |
| | GEM | | 46 | 25–76 |
| | | GEMS-6† | 46 | 22–79 |
| | | GEMS | 43 | 22–57 |
| EF | | | 58 | 37–74 |
| | EFM-6† | | 54 | 25–93 |
| | EFM | | 54 | 18–103 |
| | | EFMS-6† | 53 | 31–87 |
| | | EFMS | 52 | 26–89 |
| EE | | | 57 | 29–84 |
| | | EEMS-5† | 55 | 26–89 |
| VN | | | 82 | 55–103 |
| | VNM-8† | | 79 | 60–101 |
| | VNM | | 80 | 52–98 |
| | | VNMS-6† | 74 | 39–102 |
| | | VNMS | 74 | 41–97 |

* 50 or 100 metaphases analysed.

† Number of the subculture studied. The number was omitted when the cell lines had been subcultured more than 70 times.

### RESULTS

### *Establishment of continuous cell lines*

In this study we have established continuous cell lines from human melanoma xenografts by two different procedures: by cultivation directly in monolayers or from colonies in soft agar. Interestingly, the 4 xenografts showed differences in their behaviour.

When tumour fragments and dispersed cells were seeded out in monolayers, cultures from xenograft GE were usually overgrown by normal murine cells in the 2nd–4th subculture. However, in one instance, when the melanoma cells grew more rapidly than usual in the primary culture, the murine cells disappeared and a continuous melanoma cell line, GEM, developed. The notation for the xenografts and the derived cell lines is apparent from Table I. Xenograft EF immediately gave rise to the cell line EFM, which has been described previously (Tveit *et al.*, 1980*b*). This line grew rapidly right from the start, and no contamination with murine cells was seen. In contrast, primary cultures from xenograft EE were regularly overgrown by murine fibroblasts, and no cell line could be established.

The behaviour of xenograft VN was particularly interesting. When fragments and single cells from this xenograft were grown in culture flasks, in most cases two cell populations were found growing together. The two populations were identified as human tumour cells and abnormal murine cells by chromosome and isozyme analyses, as well as by Hoechst staining. Both populations were usually present to about the 30th subculture. Thereafter, in 2 experiments a human cell line emerged, one of which, VNM, was further studied. Usually the abnormal murine cells took over and gave rise to continuous murine cell lines with a transformed phenotype. This cell line will be discussed below.

When colonies formed in soft agar were further grown in monolayers, the xenografts gave rise to cell lines (denoted by adding S to the name), which were permanent, except EEMS, which could not be subcultured for more than 6 months.

FIG. 1.—Histological sections of human melanoma xenografts. H. & E. × 400. (a) EF, (b) VN, (c) GE.
FIG. 2.—Photomicrographs of cells in tissue culture. Phase contrast. × 160. (a) EFM, (b) VNM, (c) GEM.
FIG. 3.—Colonies in soft agar (after 2 weeks' incubation). × 160. (a) EMF, (b) VNM, (c) GEM.

*Characterization of melanoma cell lines and comparison with the xenografts of origin*

*Morphology and pigmentation.*—Three of the melanoma xenografts were closely similar in histology, whereas in xenograft VN the cells contained less cytoplasm and had nuclei of a more uniform size (Fig. 1). The derived melanoma cell lines all showed individual and characteristic cytological appearance and growth pattern (Fig. 2). The EFM and EFMS cells were triangular or bipolar, grew in irregular patterns and piled up when grown to high density. The VNM cells were bipolar, often arranged in a regular parallel fashion and a variable number of living cells floated free in the medium. Interestingly, the VNMS cells, established by a different method from the same xenografted melanoma, showed a more triangular, cuboidal and irregular appearance. In the remaining cell lines (GEM, GEMS and EEMS) cuboidal cells were most prominent.

The xenograft EF and the derived cell lines EFM and EFMS showed pigmentation, detectable by microscopic inspection of stained histological sections or monolayer cultures, and electron microscopy revealed melanosomes. In none of the other xenografts or cell lines was there evidence of melanin. Prolonged treatment of the permanent cell lines EFM, VNMS and GEMS with $2 \times 10^{-7}$M $\alpha$-MSH had no effect on melanin production, growth rate or cellular morphology, in agreement with the finding that most human melanoma lines so far studied did not respond to this hormone (Fuller & Meyskens, 1979; Lotan & Lotan, 1980). However, when treated with TPA, DMSO, retinoic acid and theophylline, our cells changed morphology and developed elongated dendritic processes. No melanin appeared in the melanin-free cell lines VNMS and GEMS, though we have previously shown increased production of melanin in the cell line EFM when treated with these agents (Tveit *et al.*, 1980*b*).

*Chromosome and isozyme studies.*—The melanoma xenografts showed dissimilar chromosome numbers (Table I). Thus, xenograft GE was near-diploid, xenografts EF and EE were hyperdiploid and xenograft VN was hypotetraploid. The *in vitro* cell lines had chromosome numbers similar to the xenografts from which they were derived (Table I) though there was a tendency for a slight downward drift for 3 xenografts. In consecutive subcultures the chromosome numbers remained stable, except in the case of the GEMS line, where a slight downward drift continued.

Also, when the lactate dehydrogenase (LDH) isozyme pattern was studied, individual differences were found between the xenografts. Thus, in three of the xenografts (EF, GE and EE) Bands 3 and 4 were the most heavily stained, indicating nearly equal amounts of A and B polypeptide chains (not shown). In contrast, in xenograft VN, Bands 4 and 5 were the most heavily stained, corresponding to higher amounts of A than B chains. Again the cell lines *in vitro* showed similar patterns to the xenografts from which they were derived.

*Growth characteristics.*—Growth curves of 5 cell lines *in vitro*, established from 3 of the xenografts, are shown in Fig. 4. It is seen that the cell line EFM showed the highest growth rate, and that in the two cases where two cell lines were obtained from the same xenograft these grew at essentially the same rate. In Table II the doubling times in culture of all cell lines are given, together with the tumour volume-doubling times (Td) previously observed *in vivo* during exponential growth (Fodstad *et al.*, 1980). It can be seen that the growth rates of the cell lines are not correlated with those of the xenografts. Thus, the most fast-growing cell line *in vitro*, EFM (doubling time 18 h), which also showed the highest saturation density, was established from a xenograft with an intermediate growth rate *in vivo* (Td 6·3 days). Three more slow growing cell lines originated from two xenografts which had considerably higher growth rates *in vivo*

Fig. 4.—Growth curves for 5 melanoma cell lines *in vitro*. Symbols: ○, EFM; □, VNM; ■, VNMS; △, GEMS; ▲, GEM.

TABLE II.—*Growth characteristics of human melanoma xenografts* in vivo *and of the corresponding cell lines* in vitro*

| Xenograft | Cell line | Doubling time *In vivo*† (days) | Doubling time *In vitro* (h) | Saturation density (cells × $10^{-4}$/cm²) | PE in soft agar‡ (%) |
|---|---|---|---|---|---|
| EF | | 6·3 (5·7–7·0) | | | 12 (5–21) |
| | EFM-6 | | 32 (29–36) | | 27 (18–35) |
| | EFM | | 18 (17–21) | 12 (10–14) | 53 (35–78) |
| | EFMS-6 | | 42 (38–46) | | 28 (20–36) |
| | EFMS | | 25 (23–27) | 11 (11) | 47 (35–65) |
| VN | | 3·3 (2·9–3·4) | | | 0·7 (0·5–1·5) |
| | VNM-8 | | 37 (36–38) | | 1·0 (0·5–1·5) |
| | VNM | | 30 (26–32) | 3·5 (3·0–4·0) | 5·0 (3·5–6·5) |
| | VNMS-6 | | 33 (31–35) | | 1·1 (0·6–1·6) |
| | VNMS | | 29 (26–32) | 5·5 (4·6–6·4) | 3·2 (2·0–4·8) |
| GE | | 9·5 (9·0–9·8) | | | 0·5 (0·2–1·0) |
| | GEM-6 | | 42 (42) | | 0·9 (0·3–1·2) |
| | GEM | | 39 (36–42) | 3·2 (2·5–4·0) | 1·1 (0·5–1·5) |
| | GEMS-6 | | 39 (36–42) | | 1·0 (0·4–1·5) |
| | GEMS | | 31 (28–36) | 4·6 (4·2–5·0) | 2·0 (0·9–2·5) |
| EE | | 3·0 (2·9–3·0) | | | 10 (5–20) |
| | EES-5 | | 42 (42) | 5·0 (3·5–6·5) | 12 (6–15) |

* Averages of 2–5 experiments (ranges in parentheses).

† Tumour volume-doubling time (volume = $\pi/6 \times$ (mean diameter)$^3$).

‡ Number of colonies (> 50 cells)/Number of cells plated × 100.

than EF. Interestingly, it was found that the amount of cellular fibronectin was inversely related to the growth rate *in vivo*. Treatment of the cell lines EFM, VNMS and GEMS with the differentiating agents TPA, DMSO, retinoic acid and theophylline always decreased the growth rates (Table III), though individual differences in response were observed.

The plating efficiencies (PE) of the different xenografts and the derived cell lines differed widely (Table II). The PE of the cell lines were clearly correlated with and consistently higher than the PE of the corresponding xenografts. In the cell lines the PE in soft agar was generally highest in the lines showing low doubling times, whereas in the xenografts there was no correlation between PE and doubling times.

*Colony morphology*.—The morphology of the colonies in soft agar was characteristic for each particular xenograft, and this morphology was maintained in the *in vitro* cell lines. Thus, the xenograft VN and the human cell lines established from it gave rise to colonies of very loosely attached cells (Fig. 3), while the xenograft GE and the cell lines derived from it gave compact small colonies (Fig. 3). Xenograft EF and the corresponding cell lines EFM and EFMS gave rise to colonies of an intermediate morphology (Fig. 3). The results indicate that the cohesion between the cells differed in soft-agar colonies from different melanomas.

To study in more detail the adhesive properties of the cells, their attachment to plastic surface and their protein content were examined. The rate of attachment to and spreading on plastic surface were studied in 3 cell lines showing different colony morphology. The GEMS cell line, which had the most compact small colonies, attached and spread out as fast as normal human fibroblasts, whereas the EFM cells, and particularly the VNMS cells, which formed colonies of loosely attached cells, needed more time for both attachment and spreading. All 3 cell lines contained surface-associated and intracellular fibronectin, as revealed by immunofluorescence. The rapidly spreading cell line GEMS had about as much fibronectin as human fibroblasts, while EFM cells, and especially the slow-spreading VNMS cells,

FIG. 5.—Photomicrographs of cells in monolayer cultures stained for fibronectin by indirect immunofluorescence. ×400. Intracellular fibronectin on left and surface-associated fibronectin on right. (a) and (b) VNMS, (c) and (d) GEMS, (e) and (f) normal human fibroblasts.

TABLE III.—*Growth inhibition of human melanoma cells treated with TPA, DMSO, retinoic acid and theophylline*

| Cell line | Percentage inhibition* after treatment for 4 days with: TPA ($10^{-7}$M) | DMSO (1·5%) | Retinoic acid ($10^{-5}$M) | Theophylline (1 mM) |
|---|---|---|---|---|
| EFM | 85 (75–95) | 72 (62–82) | 25 (10–40) | 40 (36–54) |
| VNMS | 71 (58–83) | 75 (62–88) | 42 (39–44) | 70 (50–90) |
| GEMS | 39 (26–52) | 36 (32–39) | 15 (5–25) | 56 (47–64) |

* Percentage inhibition was calculated as: 100 (1–T/C), where T and C are the numbers of cells in treated and control cultures, respectively.

The data represent the average of 6 cultures in 2 experiments (ranges in parentheses).

had less (Fig. 5). HeLa cells were devoid of fibronectin, as judged by this method. In agreement with these findings lacto-peroxidase-catalysed iodination ($^{25}$I) of surface proteins and autoradiography of SDS polyacrylamide gels, revealed the heavy fibronectin band of normal human fibroblasts in all 3 cell lines, which were most heavily labelled in the GEMS cells and least in the VNMS cells (Fig. 6).

*Tumorigenicity.*—Cells from 4 of the melanoma cell lines (EFM, VNM, VNMS and GEMS) were harvested and inoculated s.c. in athymic mice. Tumours formed easily from 3 of the cell lines, but the cell line VNMS did not form tumours, even when $10^7$ cells were injected.

Fig. 6.—Autoradiograms of SDS gels of cellular proteins labelled by lactoperoxidase-catalysed iodination with $^{125}$I. Slab-gel electrophoresis with a 4·5% stacking gel and a 7% separation gel. The gels were dried and autoradiographed. Three melanoma cell lines were examined. (a) EFM cells; (b) VNMS; (c) GEMS; (d) human fibroblasts. The arrow indicates the position of fibronectin.

*Transformation of murine cells by the human melanoma xenograft*

It was mentioned above that xenograft VN gave rise to a continuous murine cell line with unusual properties. Thus, the cells grew in an irregular pattern (Fig. 7) and were heteroploid (modal chromosome number 61, range 25–82). Furthermore, they formed colonies in soft agar (PE 0·2% in the 6th subculture and 1% in the 70th) and readily produced transplantable tumours in athymic mice. In ordinary BALB/c mice, however, the take rate was low (1/10). The tumours had a sarcomatous histology (Fig. 7B) and were shown to be of murine origin, both by the LDH isoenzyme pattern (murine bands 4 and 5) and the chromosome constitution (not shown).

Chromosome analyses of the VN xenograft and of the primary cultures revealed no evidence of transformed murine cells, indicating that the transformation had occurred *in vitro*. Consecutive studies of the *in vitro* cultures showed that the heteroploidy of the murine cells did not appear until in the 4th subculture. In control experiments cultivated fibroblasts from athymic mice had diploid chromosomes.

To study in more detail the relationship between the abnormal murine cells and the malignant human cells originating from xenograft VN, we took advantage of the fact that diphtheria toxin in appropriate concentrations will kill human cells but is non-toxic to murine cells. A series of different subcultures were treated with 25 ng/ml of diphtheria toxin. No cell line could be established from the first subcultures, but continuous murine cell lines

FIG. 7.—Photomicrographs of the malignant murine cell line established from xenograft VN (a) Cells in monolayer culture. Phase contrast × 160. (b) Histological section of tumour formed in athymic mice. H. & E. × 400.

did emerge in the 4th and later subcultures. These murine cells showed the same malignant characteristics as the spontaneous murine cell lines. It thus appears that the transformation of the murine cells occurred during the early subcultures and that somehow the presence of the human cells was necessary for the transformation.

### DISCUSSION

Continuous human cell lines have been established from a variety of malignancies, including primary and metastatic malignant melanomas (Romsdahl & Hsu, 1972; Gerner *et al.*, 1975; Giovanella *et al.*, 1976). It is difficult to tell whether these cell lines reflect the properties of the tumour cells *in vivo* by direct comparison with the patients' tumours. However, evidence is accumulating that human xenografts grown in immune-deprived animals largely reflect the properties of the parent tumours, though kinetic differences have been observed (Steel & Peckham, 1980). Since such serially heterotransplanted tumours represent a bank of easily available tumour tissue and permit repeated studies of tumour cells from the same patient, they greatly facilitate comparisons between the properties of established *in vitro* cell lines and the tumour cells of origin.

The present results show that, as expected, the melanoma cell lines, like the xenografts from which they were derived, have distinct individual properties. The comparisons of the cell lines with the corresponding xenografts demonstrate that the properties of the cell lines largely reflect those of the tumours of origin. Thus, the ultrastructural picture and the melanin pigmentation were the same *in vitro* and *in vivo*, in agreement with the findings of Foa & Aubert (1977). Also the isozyme patterns of the xenografts were retained in the cultured cell lines. The colonies formed in soft agar from the xenografts and from the derived cell lines were closely similar in respect of size and compactness. Likewise, the chromosome constitution was, by and large, retained on cultivation, though a slight downward drift in chromosome number was found during the establishment of some of the cell lines.

However, some of the *in vivo* characteristics were not retained. Importantly, the growth rates and the saturation densities of the cell lines in monolayer cultures, as well as the PE in soft agar, showed no correlation with the early growth rates of the melanomas *in vivo*. The reason for this discrepancy is not clear. One possibility is that the *in vitro* conditions influence the growth of tumour cells from different tumours to varying extents. Unexpectedly, one of the cell lines, VNMS, established *via* soft agar, did not form tumours when injected back into athymic mice. Apparently, the genetic information necessary for tumorigenicity had been lost or suppressed during the establishment in culture. It is noteworthy that in this cell line the chemosensitivity did not reflect that of the parent xenograft, in contrast to the situation with the other cell lines (Tveit *et al.*, 1981). The data emphasize that extrapolations from continuous cell lines in tissue culture to the parent tumour cells *in vivo* are not always valid.

It is a general view that fibronectin plays a central role in cell adhesion (Hynes, 1979; Kleinman *et al.*, 1981). The present finding that the amounts of fibronectin correlated with the compactness of the colonies formed in soft agar and the rapidity of attachment and spreading on a plastic surface, indicates that fibronectin may be responsible for the different degrees of compactness seen in soft-agar colonies. It has been speculated whether the amount of fibronectin on the surface of tumour cells correlates to any *in vivo* characteristics; *e.g.* stage of disease, growth rate or metastatic properties (Lloyd *et al.*, 1979). Our study shows that in the 3 tumour cell lines studied the amount of fibronectin indeed correlated with the growth rates *in vivo*, as the slowest growing xenograft (GE) gave rise to a cell line *in vitro* with high amounts of fibronectin, whilst the xenograft with the shortest doubling time (VN) gave rise to melanoma cells with least fibronectin.

A difficulty in establishing cell lines from human tumours is that most frequently stromal fibroblasts grow along with the malignant cell population and tend to overgrow the culture. To eliminate these normal cells several methods have been used, such as differential centrifugation, differential trypsinization, physical removal of colonies and the use of special culture media (Fogh, 1975). Here we have introduced an additional procedure for the elimination of fibroblasts. We have taken advantage of the fact that, in solid tumours, only malginant cells can form colonies in semi-solid medium. Thus, we have cultivated in monolayers colonies formed in soft agar. This procedure for establishing cell lines free of normal cells can also be applied directly to patients' biopsies, and we have obtained several pure malignant cell populations from patients' melanomas.

We have reported (Tveit *et al.*, 1980*a*) that when a human embryonal carcinoma xenograft was grown in tissue culture, a transformed murine cell line appeared. This abnormal heteroploid murine cell line grew in soft agar, but failed to form tumours in athymic mice. In contrast, the murine cell line which appeared in the present study upon cultivation of the melanoma xenograft VN, was clearly malignant as it gave sarcomas on injection into mice. Goldenberg and Pavia have reported that when human xenografts of 3 different histological types were grown in culture, malignant murine cells consistently emerged, capable of forming sarcomas when injected into nude mice (Pavia & Goldenberg, 1979; Goldenberg & Pavia, 1981). On this basis they suggested that such transformation may be a general phenomenon when xenografts are cultured *in vitro*. Our results do not support this view, as only one of the 4 melanoma xenografts studied gave rise to transformed murine cells.

It is apparent from this work that the malignant transformation of murine cells took place during the first subcultures. It is unlikely that spontaneous transformation can account for the transformed murine cells here observed, since they were found in all experiments involving xenografts VN and never when other melanoma xenografts or skin fibroblasts from nude mice were grown *in vitro*. Furthermore, when the human cells were killed with diphtheria toxin, it was concluded that the presence of human cells during the first 4 subcultures was necessary for the establishment of the malignant murine cell lines. The data suggest that the malignant transformation of murine stromal cells is caused by a transforming factor produced by the human melanoma cells during the first weeks of *in vitro* cultivation. This was supported by the finding that medium from the permanent melanoma cell line VNM, established from the same xenograft, consistently stimulated the growth of nude mice fibroblasts in monolayer cultures, far more than did medium from control cells. Moreover, in two instances murine fibroblasts grown in this conditioned medium formed colonies in soft agar (PE 0·2%). However, further work is clearly needed to elucidate the mechanism of this transformation.

The authors are indebted to Drs Ø. Fodstad and S. Olsnes for stimulating discussions, to Dr A. Sundan for help with the electrophoreses and to L. Næss for skilful technical assistance.

### REFERENCES

Courtenay, V. D. & Mills, J. (1978) An *in vitro* colony assay for human tumours grown in immune-suppressed mice and treated *in vivo* with cytotoxic agents. *Br. J. Cancer*, **37**, 261.

Drewinko, B., Loo, T. L., Brown, B., Gottlieb, J. A. & Freireich, E. J. (1976) Combination chemotherapy *in vitro* with Adriamycin: Observations of additive, antagonistic, and synergistic effects when used in two-drug combinations on cultured human lymphoma cells. *Cancer Biochem. Biophys.*, **1**, 187.

Eagle, H. & Foley, G. E. (1956) The cytotoxic action of carcinolytic agents in tissue culture. *Am. J. Med.*, **21**, 739.

Foa, C. & Aubert, C. (1977) Ultrastructural comparison between cultured and tumor cells of human malignant melanoma. *Cancer Res.*, **37**, 3957.

Fodstad, Ø., Aass, N. & Pihl, A. (1980) Assessment of tumour growth and of response to chemotherapy of human melanomas in athymic, nude mice. *Br. J. Cancer*, **41**, Suppl. IV, 146.

Fogh, J. (1975) *Human Tumor Cells* in vitro. New York: Plenum Press.

Fuller, B. B. & Meyskens, F. L. (1979) Endocrine responsiveness of normal and malignant human melanocytes in culture. *Proc. Am. Assoc. Cancer Res.*, **20**, 93.

Gerner, R. E., Kitamura, H. & Moore, G. E. (1975) Studies of tumor cell lines derived from patients with malignant melanoma. *Oncology*, **31**, 31.

Giovanella, B. C., Stehlin, J. S., Santamaria, C. & 6 others (1976) Human neoplastic and normal cells in tissue culture. I. Cell lines derived from malignant melanomas and normal melanocytes. *J. Natl Cancer Inst.*, **56**, 1131.

Goldenberg, D. M. & Pavia, R. A. (1981) Malignant potential of murine stromal cells after transplantation of human tumors into nude mice. *Science*, **212**, 65.

HYNES, R. O. (1979) Proteins and glycoproteins. In *Surfaces of Normal and Malignant Cells.* Ed. Hynes. New York: John Wiley & Sons. p. 103.

KLEINMAN, H. K., KLEBE, R. J. & MARTIN, G. R. (1981) Role of collagenous matrices in the adhesion and growth of cells. *J. Cell Biol.*, **88**, 473.

LAURILA, P., VIRTANEN, I., WARTIOVAARA, J. & STENMAN, S. (1978) Fluorescent antibodies and lectins stain intracellular structures in fixed cells treated with nonionic detergent. *J. Histochem. Cytochem.*, **26**, 251.

LLOYD, K. O., TRAVASSOS, L. R., TAKAHASHI, T. & OLD, L. J. (1979) Cell surface glycoproteins of human tumor cell lines: Unusual characteristics of malignant melanoma. *J. Natl Cancer Inst.*, **63**, 623.

LOTAN, R. & LOTAN, D. (1980) Stimulation of melanogenesis in a human melanoma cell line by retinoids. *Cancer Res.*, **40**, 3345.

MOSER, F. G., DORMAN, B. P. & RUDDLE, F. H. (1975) Mouse–human heterokaryon analysis with a 33258 Hoechst–Giemsa technique. *J. Cell Biol.*, **66**, 676.

PAVIA, R. A. & GOLDENBERG, D. M. (1979) Malignant transformation of murine fibroblasts propagated from a human cervical squamous cancer xenografted in nude mice. *In Vitro*, **15**, 227.

PETTERSEN, E. O., OFTEBRO, R. & BRUSTAD, T. (1974) Survival after X-irradiation of extremely hypoxic human cells cultured *in vitro*. *Int. J. Radiat. Biol.*, **26**, 305.

ROMSDAHL, M. M. & HSU, T. C. (1972) Establishment and characterization of human malignant melanoma cell lines grown *in vitro*. In *Pigmentation. Its Genesis and Biologic Control.* Ed. Riley. New York: Appleton-Century-Crofts. p. 461.

STEEL, G. G. & PECKHAM, M. J. (1980) Human tumour xenografts: A critical appraisal. *Br. J. Cancer*, **41**, Suppl. IV, 133.

TVEIT, K. M., FODSTAD, Ø., BROGGER, A. & OLSNES, S. (1980*a*) Human embryonal carcinoma grown in athymic mice and *in vitro*. *Cancer Res.*, **40**, 949.

TVEIT, K. M., FODSTAD, Ø., JOHANNESSEN, J. V. & OLSNES, S. (1980*b*) A human melanoma cell line established from xenograft in athymic mice. *Br. J. Cancer*, **41**, 724.

TVEIT, K. M., FODSTAD, Ø. & PIHL, A. (1981) The usefulness of human tumour cell lines in the study of chemosensitivity. A study of malignant melanomas. *Int. J. Cancer*, **28**, 403.

WHITTAKER, J. R. (1963) Changes in melanogenesis during the dedifferentiation of chick retinal pigment cells in cell culture. *Dev. Biol.*, **8**, 99.